# Supplementary material for: Enabling accurate and early detection of recently emerged SARS-CoV-2 variants of concern in wastewater
Source: Nat Commun. 2023 May 17;14:2834. doi: 10.1038/s41467-023-38184-3 (PMC10191095; doi:10.1038/s41467-023-38184-3)
Supplement: Supplementary file 1 — Supplementary information [file 41467_2023_38184_MOESM1_ESM.pdf]

Supplementary materials

Supplementary Table 1. Wastewater treatment plants sampled, abbreviations, average flow rates, service populations, and geographic service areas.

Wastewater treatment plant	Abbreviation	Flowrate, MGD (AVG ± SD)	Population	Average gal/cap/day	Area, square miles
69th Street	69	80.03 ± 21.77	551,150	145	96.72
Almeda Sims	AS	13.76 ± 11.93	117,968	117	54.93
Beltway	BW	6.93 ± 4.70	70,900	98	9.76
Cedar Bayou	CD	0.78 ± 0.42	1,722	453	3.27
Chocolate Bayou	CB	4.03 ± 4.19	37,359	108	14.61
Clinton Park	CP	0.69 ± 0.81	3,825	180	4.14
Easthaven	EH	1.89 ± 1.85	16,030	118	4.78
FWSD#23	23	3.09 ± 2.52	40,689	76	15.14
Forest Cove	FC	0.28 ± 0.13	4,170	67	2.73
Greenridge	GR	3.06 ± 3.06	28,742	106	6.87
Homestead	HO	1.58 ± 1.53	9,375	169	6.12
Imperial Valley	IV	1.75 ± 0.70	16,804	104	2.22
Intercontinental Airport	IA	1.91 ± 0.63	2,408	793	38.73
Keegans Bayou	KB	14.25 ± 10.31	124,000	115	13.78
Kingwood Central	KW	3.49 ± 1.46	52,055	67	23.04
Kingwood West	MG	0.61 ± 0.20	2,589	236	2.6
MUD#203	203	0.38 ± 0.12	4,010	95	2.57
Metro Central	MC	1.99 ± 1.64	20,161	99	9.86
Northbelt	NO	2.37 ± 1.49	12,892	184	15.79
Northeast	NE	3.88 ± 4.25	33,102	117	14.41
Northgate	NG	2.75 ± 1.03	19,867	138	3.6
Northwest	NW	9.99 ± 5.84	95,600	104	22.62
Park Ten	PT	0.62 ± 0.31	5,497	113	2.19
Sagemont	SG	4.52 ± 3.49	20,608	219	5.9

Sims Bayou South*	SS	23.93 ± 18.22	109,414	219	47.84
Sims Bayou North*	SB	8.22 ± 7.54	109,414	75	47.84
Southeast	SE	4.88 ± 4.85	32,485	150	9.06
Southwest	SW	37.59 ± 26.39	293,227	128	38.72
Tidwell Timbers	TT	0.11 ± 0.06	1,133	97	0.57
Turkey Creek	TC	7.00 ± 4.85	59,188	118	10.46
Upper Brays	UB	10.33 ± 7.44	97,918	105	12.81
WCID#111	111	2.24 ± 0.28	20,920	107	3.35
WCID#47	47	3.36 ± 2.28	33,645	100	6.27
WCID#76	76	0.37 ± 0.22	976	379	0.5
West District	WD	10.06 ± 6.62	85,129	118	17.86
West Lake	WL	0.20 ± 0.07	600	333	0.53
Westway	WW	0.40 ± 0.18	3,623	110	0.99
White Oak	WO	1.84 ± 0.91	20,758	89	3.31
Willowbrook	WB	1.28 ± 0.52	8,610	149	3.01
**TOTAL**		**272.23 ± 159.04**	**2,168,563**	**162 ± 133 (AVG ± STDEV)**	**532**

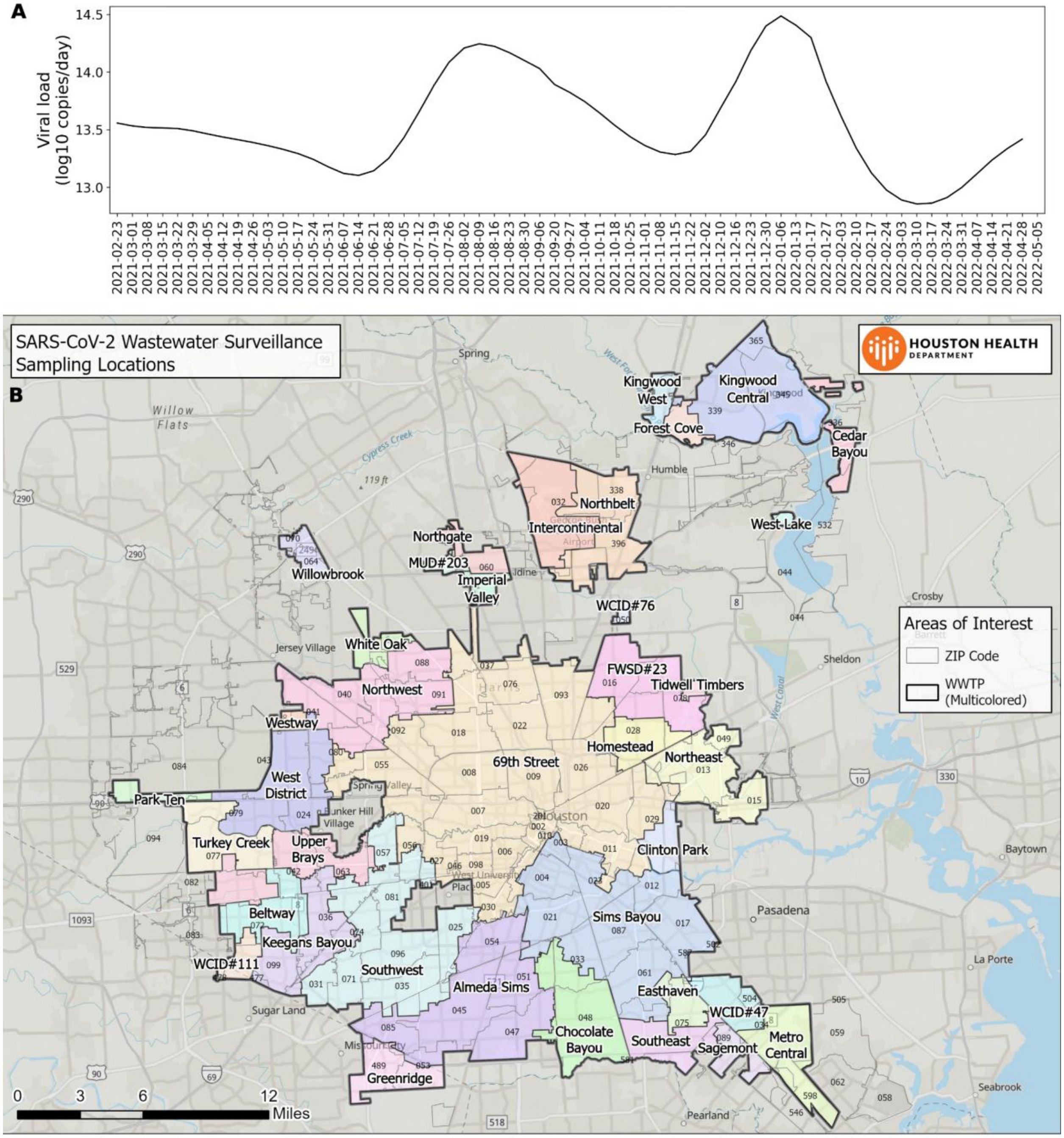

Supplementary Figure 1. A. Estimated viral load in Houston wastewater in log-scale of viral copies per day. Trend in viral load matches up with the variant associated waves of infection. **B.** Map of the sewersheds and wastewater treatment plants in the city of Houston.

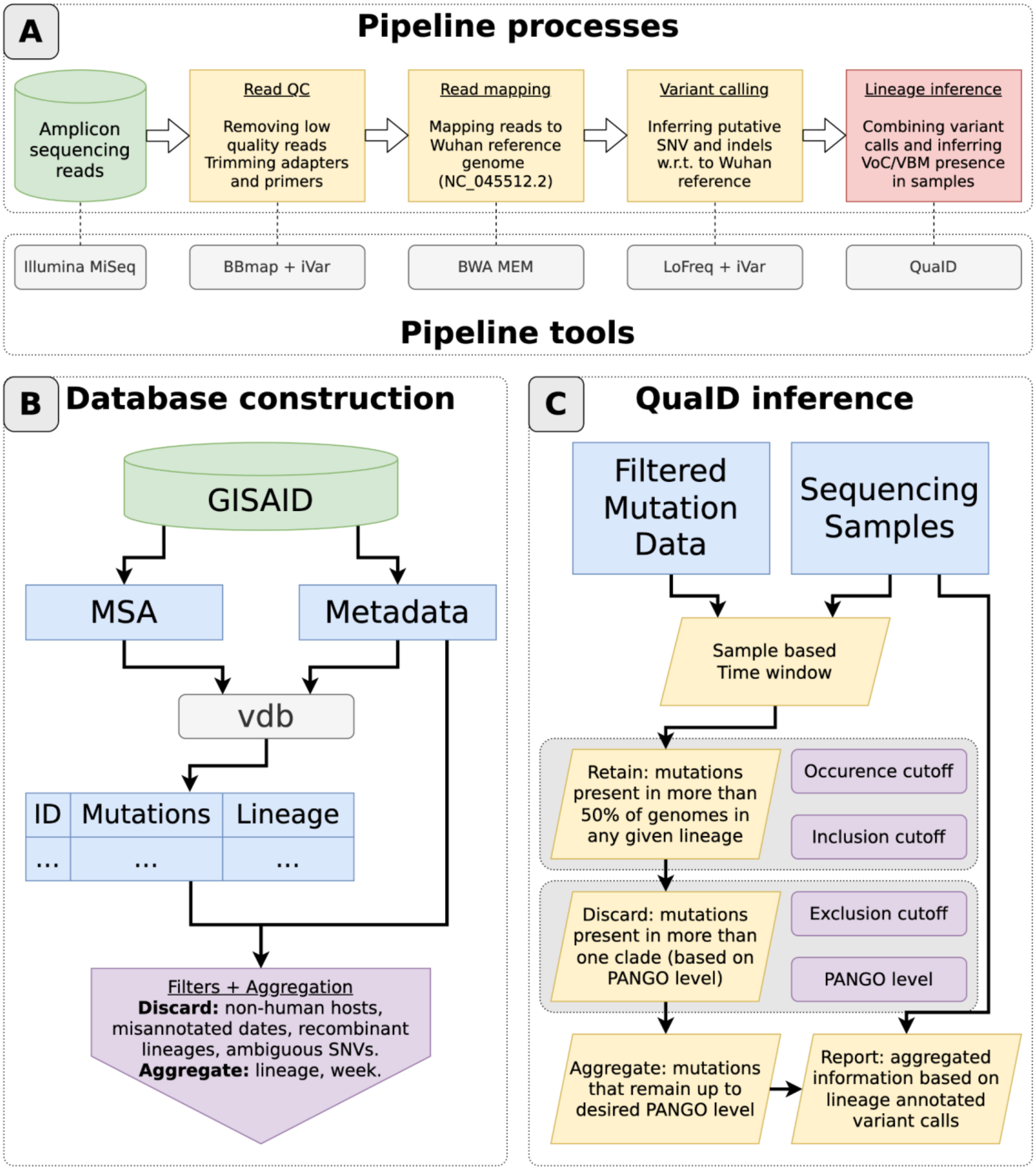

Supplementary Figure 2. Overview of QuaID computational approach. A. Overview of the complete sequencing data processing pipeline employed in the analysis of the Houston wastewater SARS-CoV-2 sequencing data. **B.** Schematics overview of the mutation database construction and sanitation used by QuaID. MSA and metadata obtained from GISAID are first pre-processed with vdb to extract mutations, and then custom Python code is used to perform additional filtering and aggregation procedures. **C.** QuaID VoC/VBM inference process overview. Parameters that affect described subroutines (yellow parallelograms) are provided in the purple rounded rectangles.

Supplementary Table 2. Sequencing protocol details including library prep kit, sequencing kit, primer panel, RNA extract template volume, and cycling recipe used for each batch of weekly wastewater samples.

Sample Date	Library prep kit	Sequencing Kit	Primer panel used	Template volume for cDNA prep	Cycling Recipe
2/23/2021 - 4/6/2021	illumina DNA prep Kit (20018705)	Kit V3-150cycle (MS-102-3001)	ARTIC V3	11 uL	151+10+10+ 151
4/13/2021 - 7/20/2021	illumina DNA prep Kit (20018705)	Kit V3-600cycle (MS-102-3003)	ARTIC V3	11 uL	151+10+10+ 151
7/27/21	illumina CovidSeq assay (20049393, 20051772)	micro Kit V2-300cycle (MS-103-1002)	ARTIC V3	8.5 uL	151+10+10+ 151
8/3/2021 - 12/14/2021	NebNext kit (neb #E7650S/L)	micro Kit V2-300cycle (MS-103-1002)	ARTIC V3	8.0 uL	151+8+8+15 1
12/21/2021 - 2/22/2022	NebNext VSS Kit (NEB #E7658S/L)	micro Kit V2-300cycle (MS-103-1002)	NEBNext VarSkip Short SARS-CoV-2 Primer	8.0 uL	151+8+8+15 1
3/1/2022 - 5/3/2022	NebNext VSS Kit (NEB #E7658S/L)	NextSeq kit-150cycle (20024907)	NEBNext VarSkip Short 2 SARS-CoV-2 Primer	8.0 uL	151+8+8+15 1

Supplementary Table 3. Description of output columns for QuaID output reports.

Output column	Description
Date	Sample date
Plant	Sample (WWTP) name
Total AF	Sum of the allele frequencies of detected QU mutations (a floating-point value in the range [0., Total QU count])
Total QU count	Count of detected QU mutations (i.e. the size of the set intersection between mutations designated as QU for the variant, and mutations occuring in a sample)
# QU possible	Total number of possible QU mutations for the given variant (if the selected time window in GISAID contains no sequences from the given variant or the inclusion/exclusion thresholds are set too stringently this value can be 0)

Fraction covered	Fraction of QU mutation sites with non-zero coverage (flanking coverage for indels). Note that this only checks the coverage of the site itself, and hence does not guarantee that a mutation was called (e.g. site with non-zero coverage, but less than 5x coverage or site with allele frequency below 0.02).
WHO name	WHO name for the considered VoC (e.g. Delta) or PANGO lineage name (e.g. BA.5)

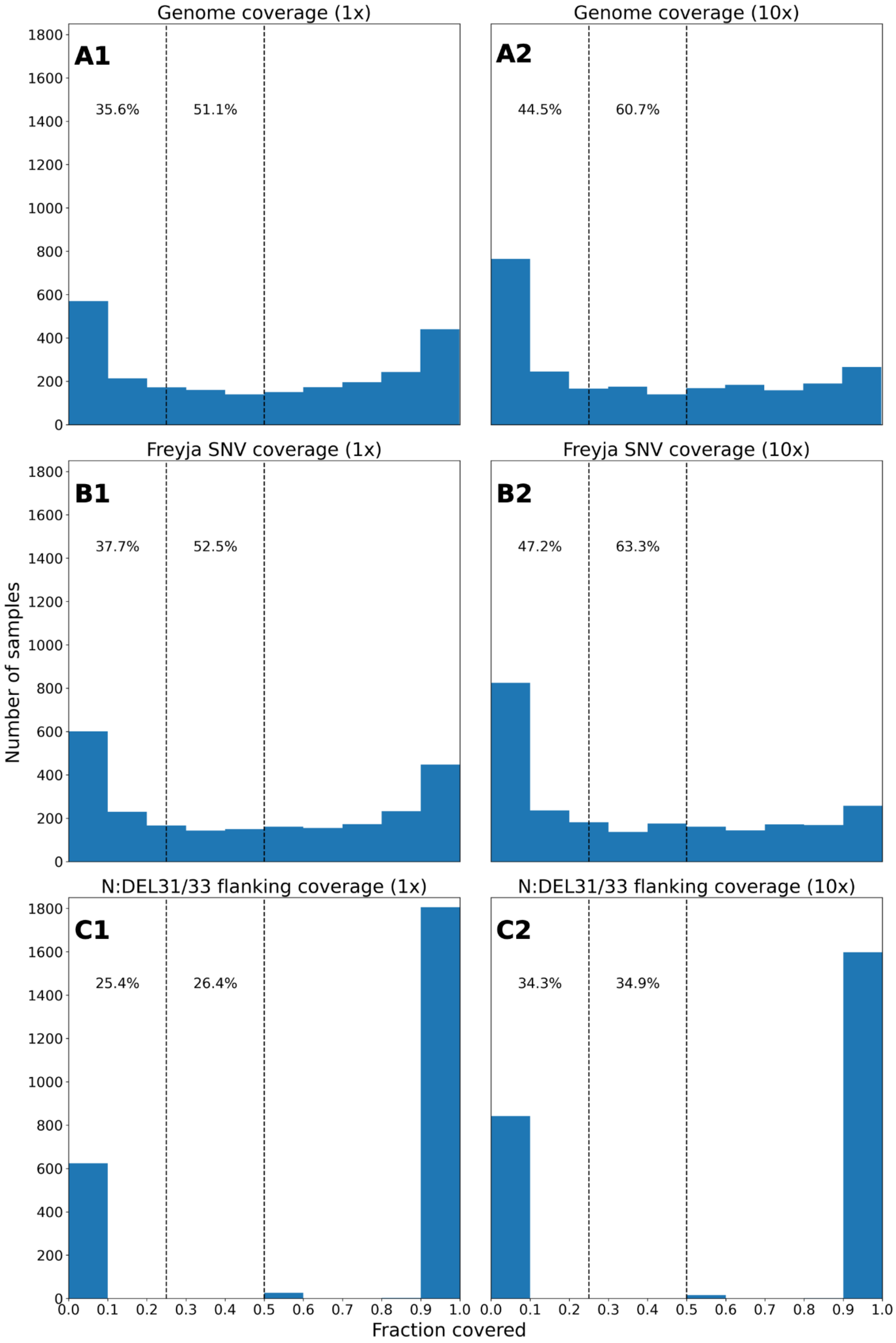

Genome coverage (1x)
Genome coverage (10x)
A1
A2
35.6%
51.1%
44.5%
60.7%
Freyja SNV coverage (1x)
Freyja SNV coverage (10x)
B1
B2
37.7%
52.5%
47.2%
63.3%
N:DEL31/33 flanking coverage (1x)
N:DEL31/33 flanking coverage (10x)
C1
C2
25.4%
26.4%
34.3%
34.9%
Number of samples
Fraction covered
0
200
400
600
800
1000
1200
1400
1600
1800
0.0
0.1
0.2
0.3
0.4
0.5
0.6
0.7
0.8
0.9
1.0

Supplementary Figure 3. A1, 2. Histogram of the coverage breadth statistics with respect to the Wuhan reference genome. Cumulative percentage of samples with coverage breadth up to 25% (35.6%/44.5% of all samples) and 50% (51.1%/60.7% of all samples) is noted above the histogram. **B1, 2.** Histogram of the fraction of the SNVs used by Freyja to detect Omicron VoC that have coverage. Cumulative percentage of samples with coverage fraction up to 25% (37.7%/52.5% of all samples) and 50% (47.2%/63.3% of all samples) is noted above the histogram. **C1, 2.** Histogram of the fraction of the flanking positions (6 in total) for the N:DEL31/33 that have coverage. Cumulative percentage of samples with coverage fraction up to 25% (25.4%/26.4% of all samples) and 50% (34.3%/34.9% of all samples) is noted above the histogram.

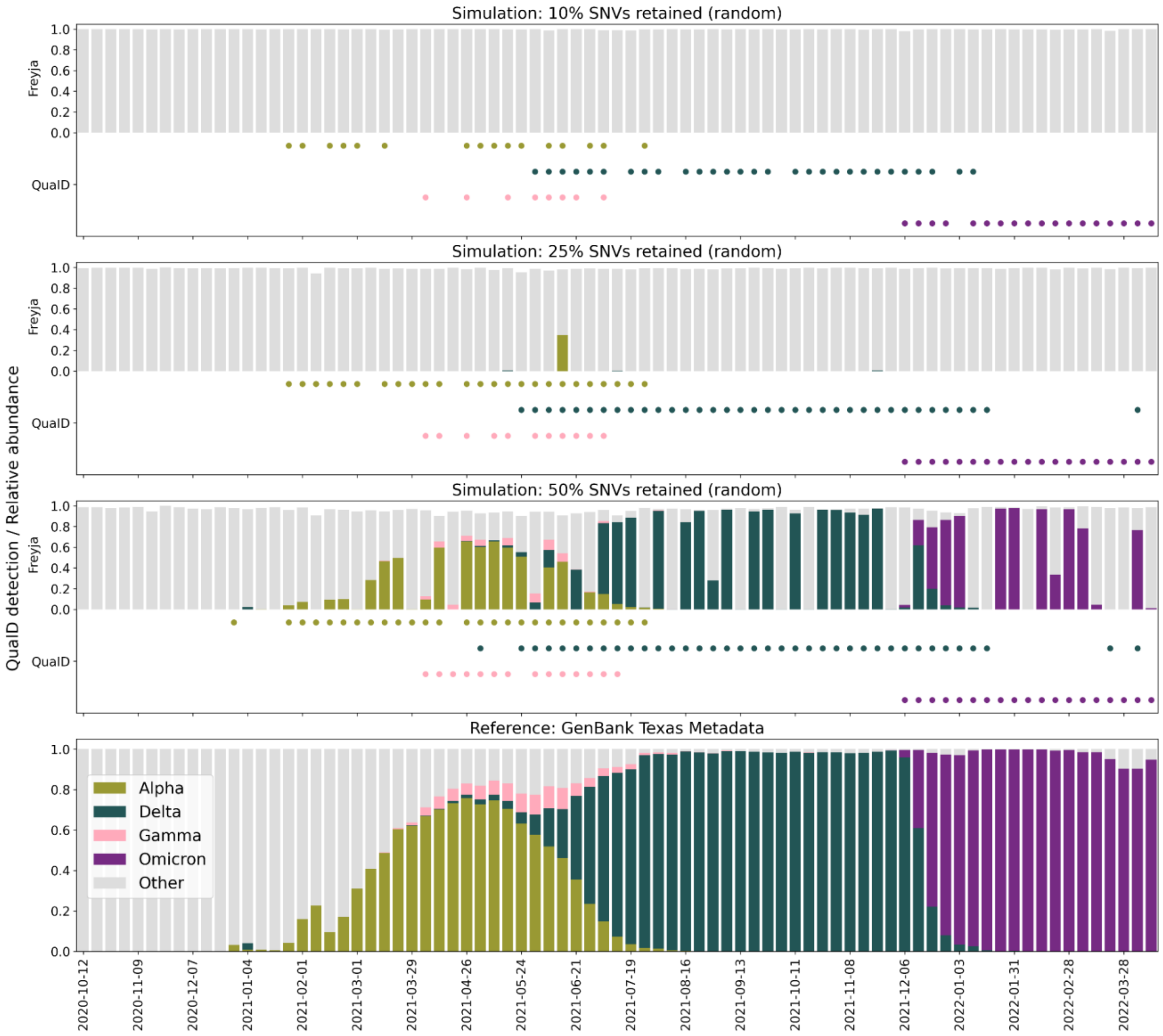

Supplementary Figure 4. (random seed 3) **A.** Freyja relative abundance estimates and QuaID detection signal on simulated data from GenBank (USA/TX) with 10% of all SNVs retained at random. Freyja is unable to detect any of the four (Alpha, Delta, Gamma, Omicron) VoCs. QuaID detection is sparse, in particular for the Gamma variant. **B.** Freyja relative abundance estimates and QuaID detection signal on simulated data from GenBank (USA/TX) with 25% of all SNVs retained at random. Freyja sparsely detects major VoCs (Delta, Omicron). QuaID detections become less sparse for all VoCs. **C.**

Freyja relative abundance estimates and QuaID detection signal on simulated data from GenBank (USA/TX) with 50% of all SNVs retained at random. Freyja detections become dense, and in some cases abundance estimates correctly reflect simulated abundance profiles. QuaID remains highly sensitive with respect to early detection. **D.** Metadata from GenBank (USA/TX) showing the fraction of genomes belonging to different VoCs for any given week. In this simulated experiment the fractions shown correspond to true relative abundances in the simulated mixture.

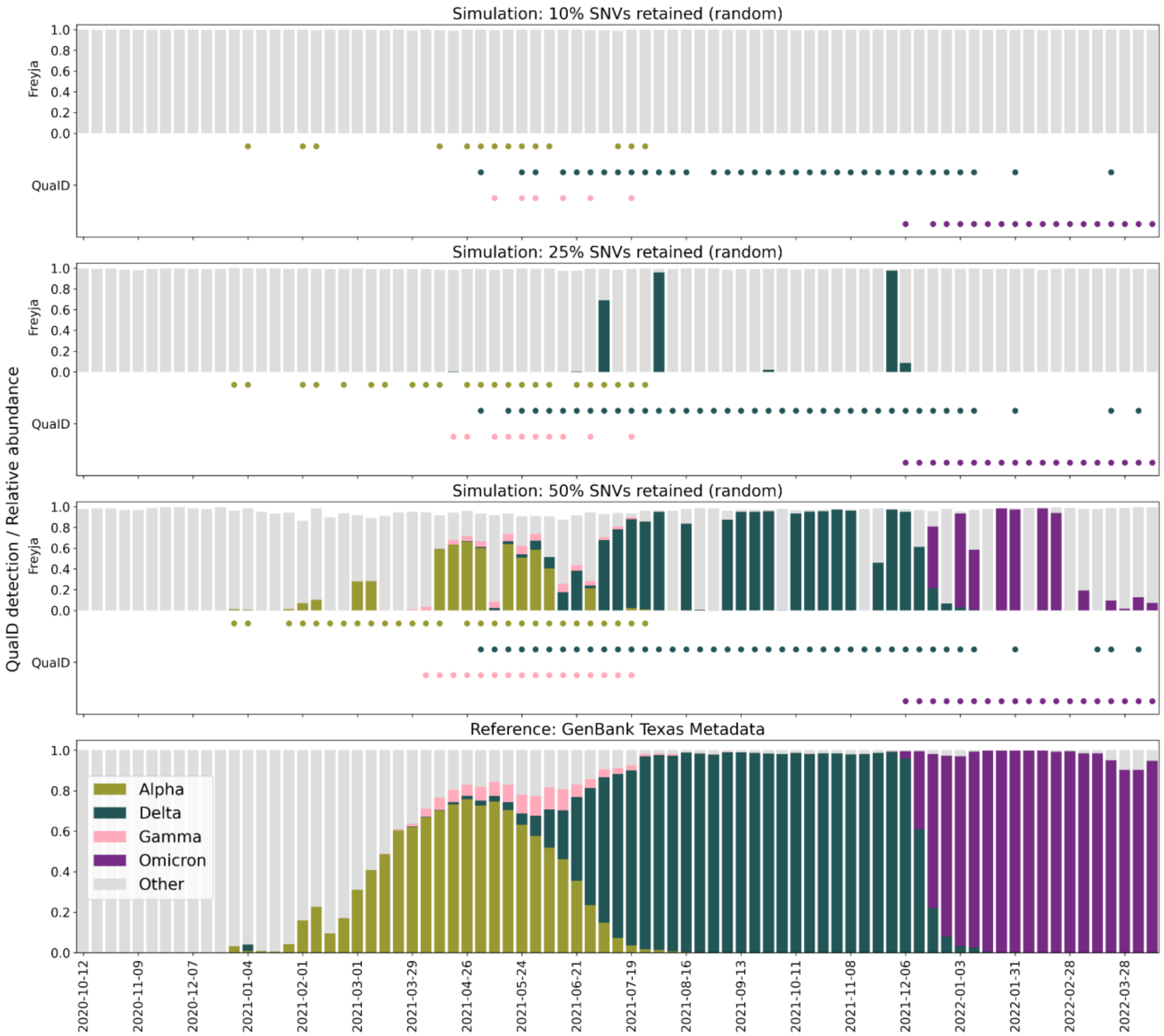

Supplementary Figure 5. (random seed 8) **A.** Freyja relative abundance estimates and QuaID detection signal on simulated data from GenBank (USA/TX) with 10% of all SNVs retained at random. Freyja is unable to detect any of the four (Alpha, Delta, Gamma, Omicron) VoCs. QuaID detection is sparse, in particular for the Gamma variant. **B.** Freyja relative abundance estimates and QuaID detection signal on simulated data from GenBank (USA/TX) with 25% of all SNVs retained at random. Freyja sparsely detects major VoCs (Delta, Omicron). QuaID detections become less sparse for all VoCs. **C.** Freyja relative abundance estimates and QuaID detection signal on simulated data from GenBank (USA/TX) with 50% of all SNVs retained at random. Freyja detections become dense, and in some cases abundance estimates correctly reflect simulated abundance profiles. QuaID remains highly sensitive with

respect to early detection. **D.** Metadata from GenBank (USA/TX) showing the fraction of genomes belonging to different VoCs for any given week. In this simulated experiment the fractions shown correspond to true relative abundances in the simulated mixture.

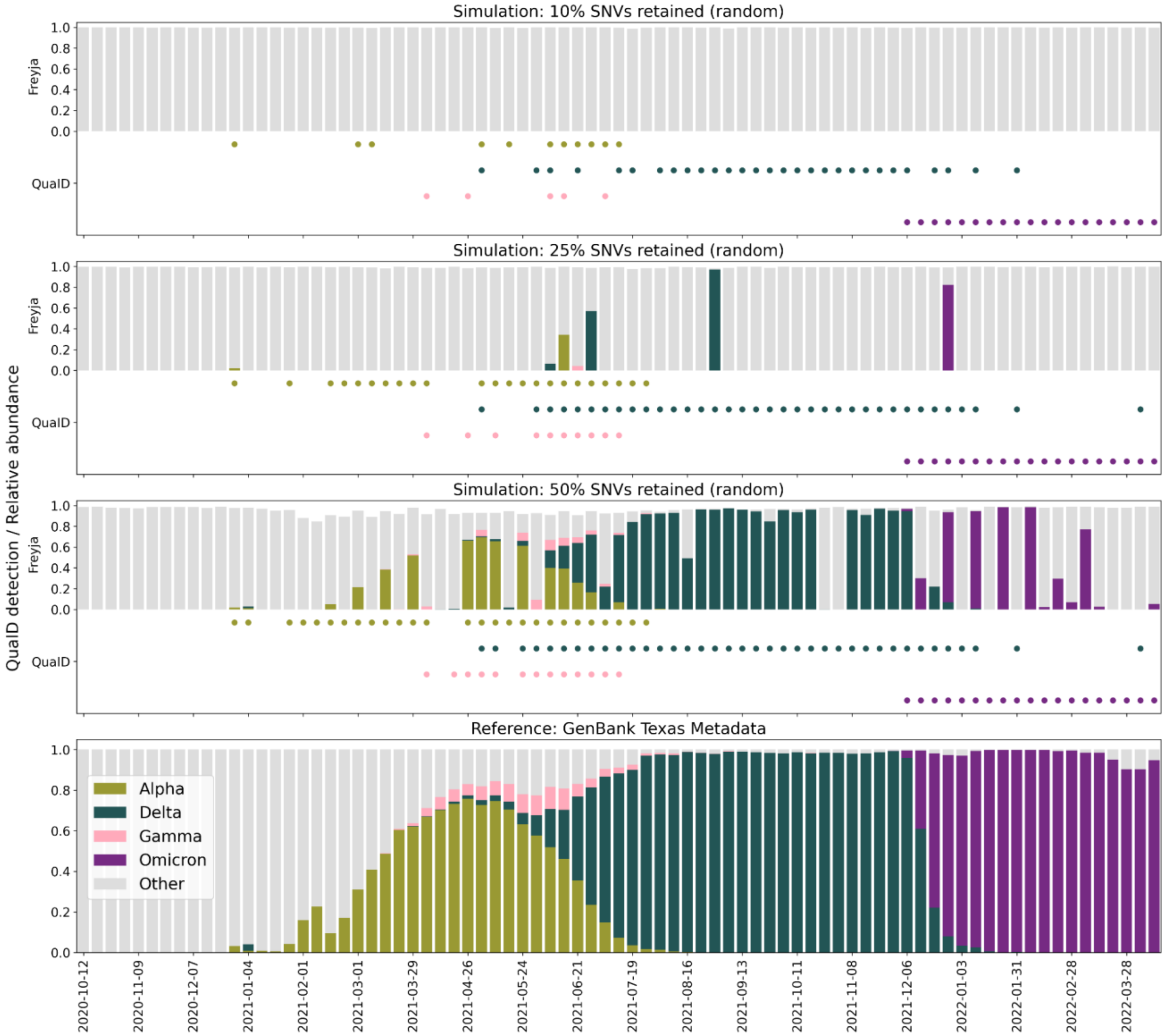

Supplementary Figure 6. (random seed 14) **A.** Freyja relative abundance estimates and QuaID detection signal on simulated data from GenBank (USA/TX) with 10% of all SNVs retained at random. Freyja is unable to detect any of the four (Alpha, Delta, Gamma, Omicron) VoCs. QuaID detection is sparse, in particular for the Gamma variant. **B.** Freyja relative abundance estimates and QuaID detection signal on simulated data from GenBank (USA/TX) with 25% of all SNVs retained at random. Freyja sparsely detects major VoCs (Delta, Omicron). QuaID detections become less sparse for all VoCs. **C.** Freyja relative abundance estimates and QuaID detection signal on simulated data from GenBank (USA/TX) with 50% of all SNVs retained at random. Freyja detections become dense, and in some cases abundance estimates correctly reflect simulated abundance profiles. QuaID remains highly sensitive with respect to early detection. **D.** Metadata from GenBank (USA/TX) showing the fraction of genomes belonging to different VoCs for any given week. In this simulated experiment the fractions shown correspond to true relative abundances in the simulated mixture.

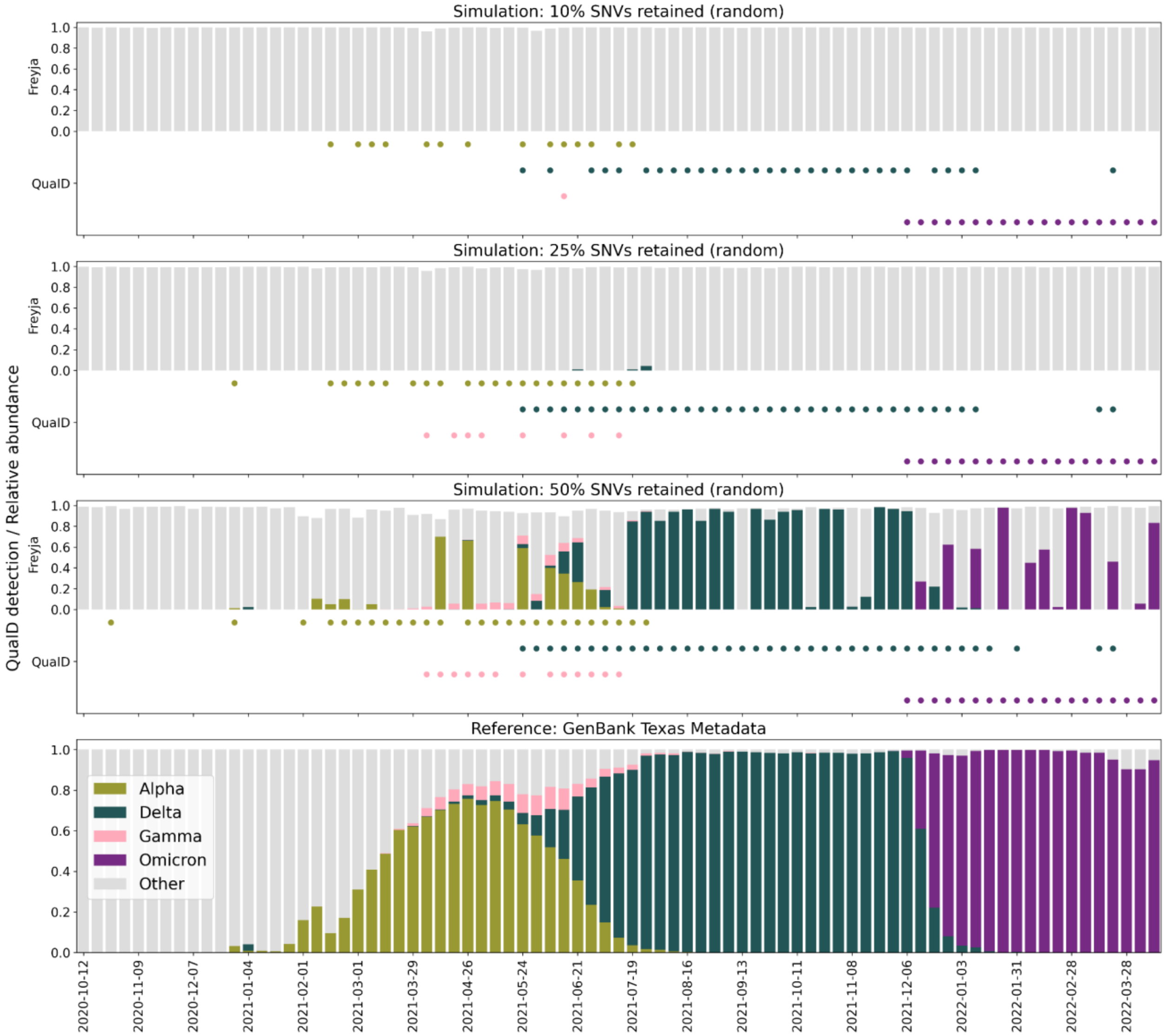

Supplementary Figure 7. (random seed 42) **A.** Freyja relative abundance estimates and QuaID detection signal on simulated data from GenBank (USA/TX) with 10% of all SNVs retained at random. Freyja is unable to detect any of the four (Alpha, Delta, Gamma, Omicron) VoCs. QuaID detection is sparse, in particular for the Gamma variant. **B.** Freyja relative abundance estimates and QuaID detection signal on simulated data from GenBank (USA/TX) with 25% of all SNVs retained at random. Freyja sparsely detects major VoCs (Delta, Omicron). QuaID detections become less sparse for all VoCs. **C.** Freyja relative abundance estimates and QuaID detection signal on simulated data from GenBank (USA/TX) with 50% of all SNVs retained at random. Freyja detections become dense, and in some cases abundance estimates correctly reflect simulated abundance profiles. QuaID remains highly sensitive with respect to early detection. **D.** Metadata from GenBank (USA/TX) showing the fraction of genomes belonging to different VoCs for any given week. In this simulated experiment the fractions shown correspond to true relative abundances in the simulated mixture.

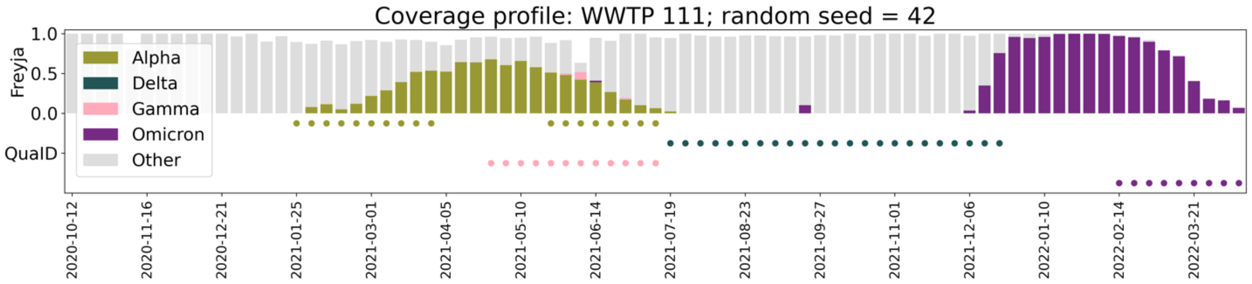

Supplementary Figure 8. (random seed 42) Freyja relative abundance estimates and QuaID detection signal on simulated data from GenBank (USA/TX) with the coverage profile based on the WWTP 111 (mutations resampled as Bernoulli trials). Freyja is unable to detect the Delta VoC, and most of the occurrences of Gamma VoC. QuaID detection suffers from drops in signal for Alpha VoC and Omicron VoC.

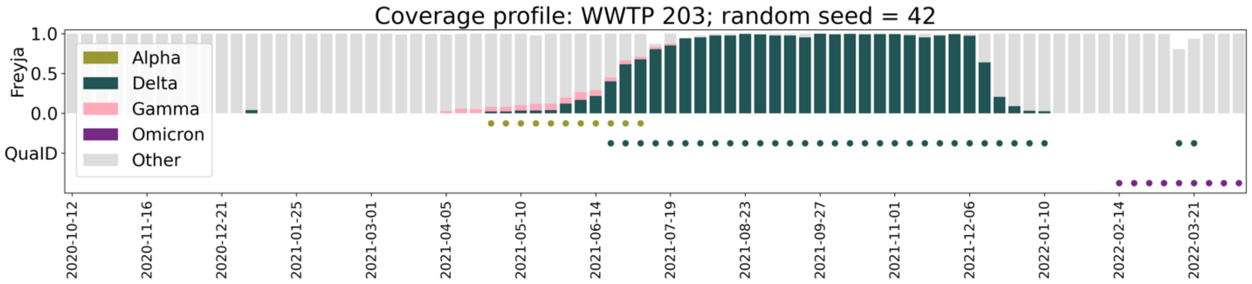

Supplementary Figure 9. (random seed 42) Freyja relative abundance estimates and QuaID detection signal on simulated data from GenBank (USA/TX) with the coverage profile based on the WWTP 203 (mutations resampled as Bernoulli trials). Freyja is unable to detect the Alpha and Omicron VoCs. QuaID detection suffers from drops in signal for Alpha and Omicron VoCs.

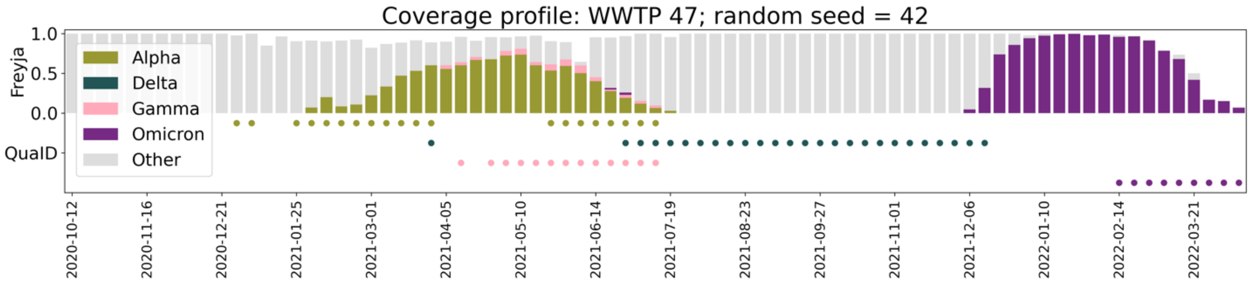

Supplementary Figure 10. (random seed 42) Freyja relative abundance estimates and QuaID detection signal on simulated data from GenBank (USA/TX) with the coverage profile based on the WWTP 47 (mutations resampled as Bernoulli trials). Freyja is unable to detect the Delta VoC. QuaID detection suffers from drops in signal for Alpha and Omicron VoCs.

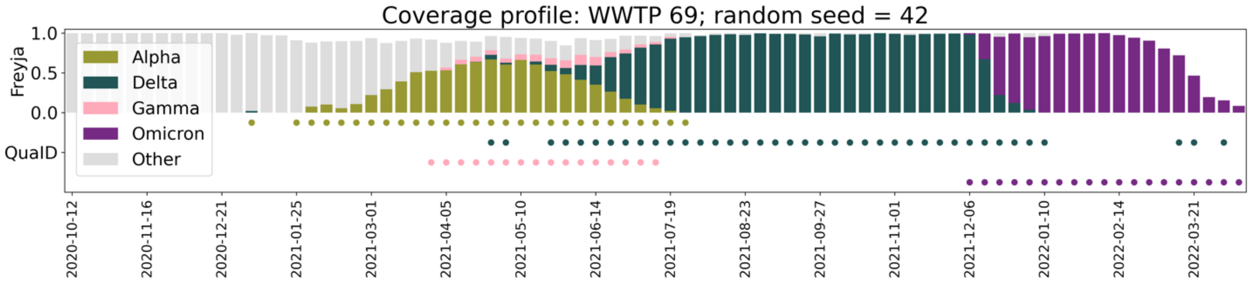

Supplementary Figure 11. (random seed 42) Freyja relative abundance estimates and QuaID detection signal on simulated data from GenBank (USA/TX) with the coverage profile based on the WWTP 69 (mutations resampled as Bernoulli trials). Both Freyja and QuaID show robust detection patterns for all VoCs. QuaID detects presence of Omicron and Gamma VoCs one week earlier than Freyja.

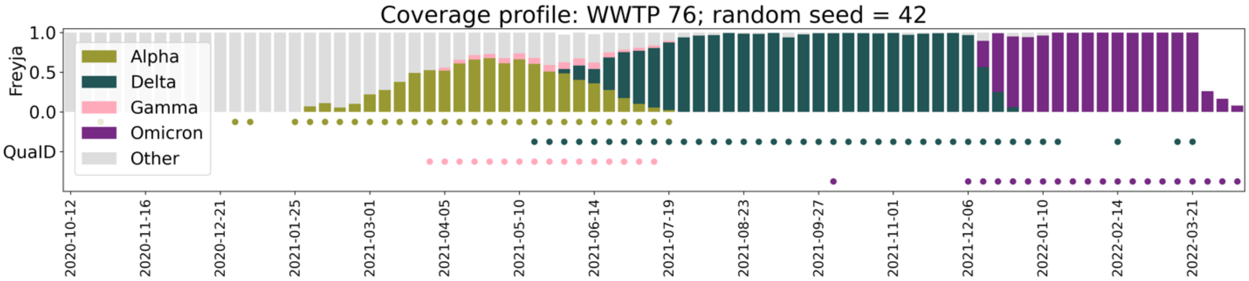

Supplementary Figure 12. (random seed 42) Freyja relative abundance estimates and QuaID detection signal on simulated data from GenBank (USA/TX) with the coverage profile based on the WWTP 76 (mutations resampled as Bernoulli trials). Both Freyja and QuaID show robust detection patterns for all VoCs. QuaID detects presence of Alpha, Delta, Gamma, and Omicron VoCs at least one week earlier than Freyja.

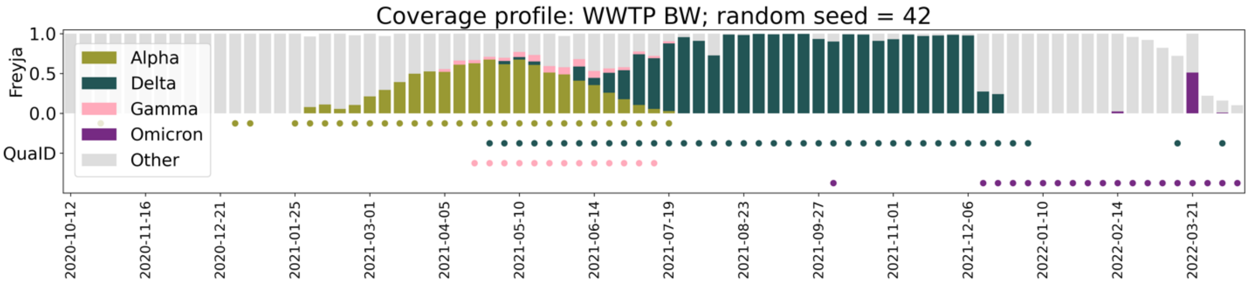

Supplementary Figure 13. (random seed 42) Freyja relative abundance estimates and QuaID detection signal on simulated data from GenBank (USA/TX) with the coverage profile based on the WWTP BW(mutations resampled as Bernoulli trials). Freyja is unable to detect the Omicron VoC. QuaID is robust for all VoCs.

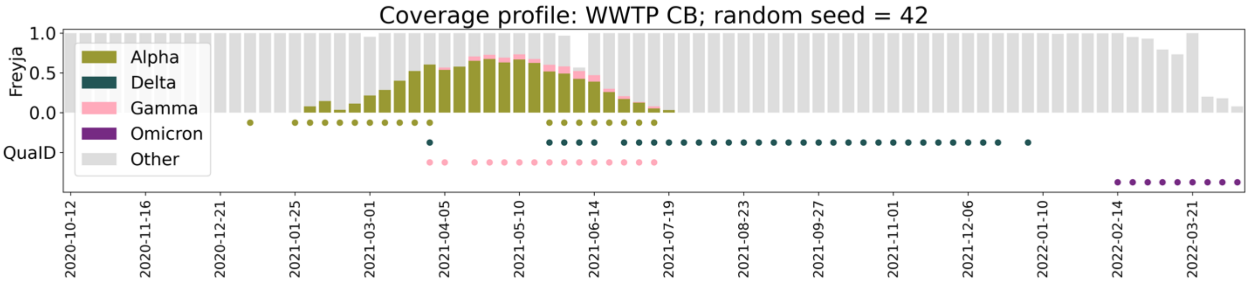

Supplementary Figure 14. (random seed 42) Freyja relative abundance estimates and QuaID detection signal on simulated data from GenBank (USA/TX) with the coverage profile based on the WWTP CB (mutations resampled as Bernoulli trials). Freyja is unable to detect the Delta and Omicron VoCs. QuaID detection suffers from drops in signal for Alpha and Omicron VoCs.

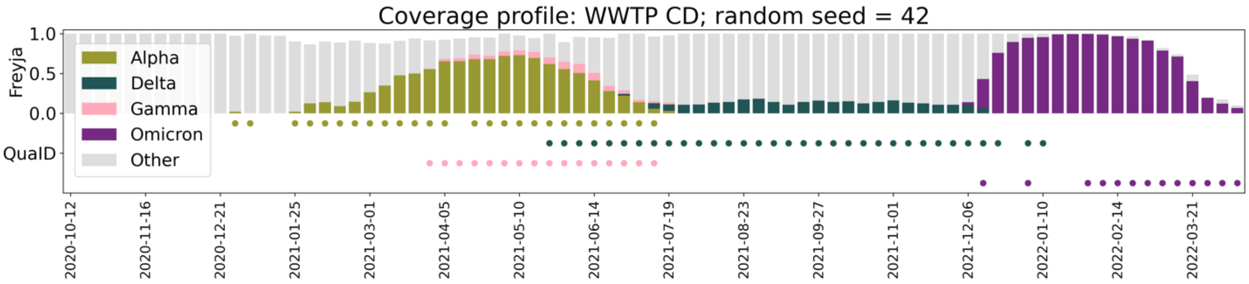

Supplementary Figure 15. (random seed 42) Freyja relative abundance estimates and QuaID detection signal on simulated data from GenBank (USA/TX) with the coverage profile based on the WWTP CD (mutations resampled as Bernoulli trials). Both Freyja and QuaID show robust detection patterns for all VoCs.

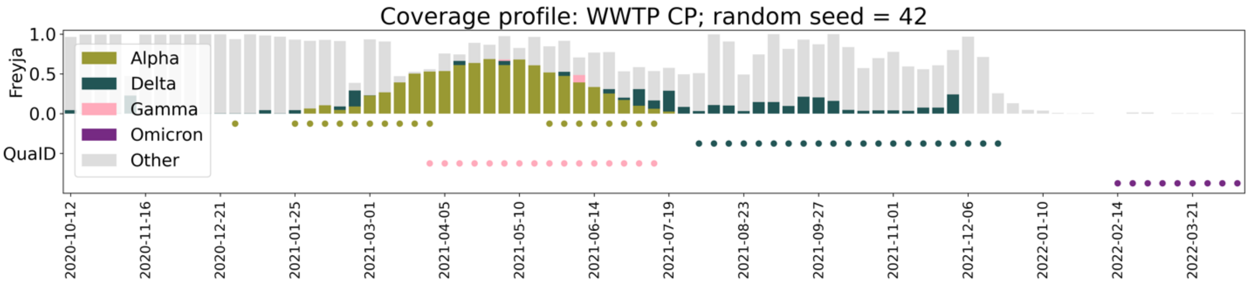

Supplementary Figure 16. (random seed 42) Freyja relative abundance estimates and QuaID detection signal on simulated data from GenBank (USA/TX) with the coverage profile based on the WWTP CP (mutations resampled as Bernoulli trials). Freyja is unable to detect the Omicron VoC. QuaID detection suffers from drops in signal for Alpha and Omicron VoCs.

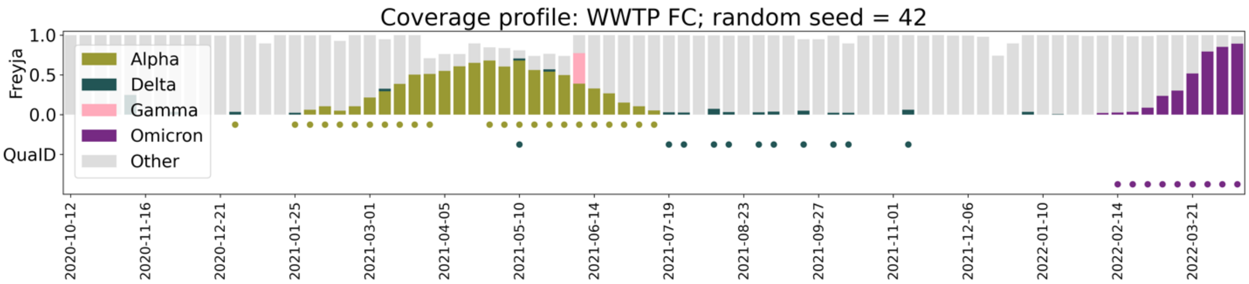

Supplementary Figure 17. (random seed 42) Freyja relative abundance estimates and QuaID detection signal on simulated data from GenBank (USA/TX) with the coverage profile based on the WWTP FC (mutations resampled as Bernoulli trials). Freyja is unable to detect the Delta and Gamma VoCs. QuaID detection suffers from drops in signal for Alpha, Gamma, Delta and Omicron VoCs.

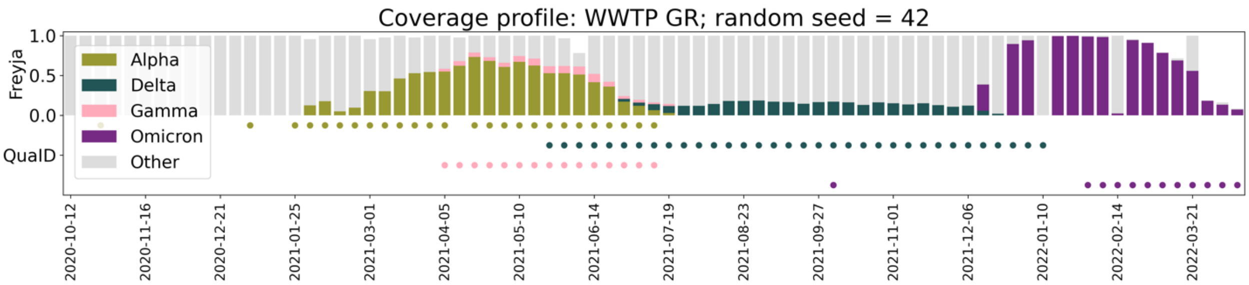

Supplementary Figure 18. (random seed 42) Freyja relative abundance estimates and QuaID detection signal on simulated data from GenBank (USA/TX) with the coverage profile based on the WWTP GR (mutations resampled as Bernoulli trials). Freyja has some drops in detection of Omicron VoC. QuaID detection suffers from drops in signal for Omicron VoC.

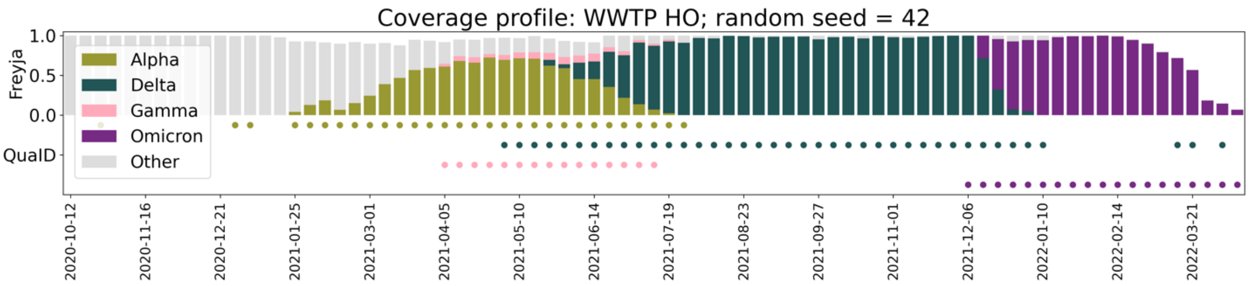

Supplementary Figure 19. (random seed 42) Freyja relative abundance estimates and QuaID detection signal on simulated data from GenBank (USA/TX) with the coverage profile based on the WWTP HO (mutations resampled as Bernoulli trials). Both Freyja and QuaID show robust detection patterns for all VoCs. QuaID detects presence of Alpha, Delta, Gamma, and Omicron VoCs at least one week earlier than Freyja.

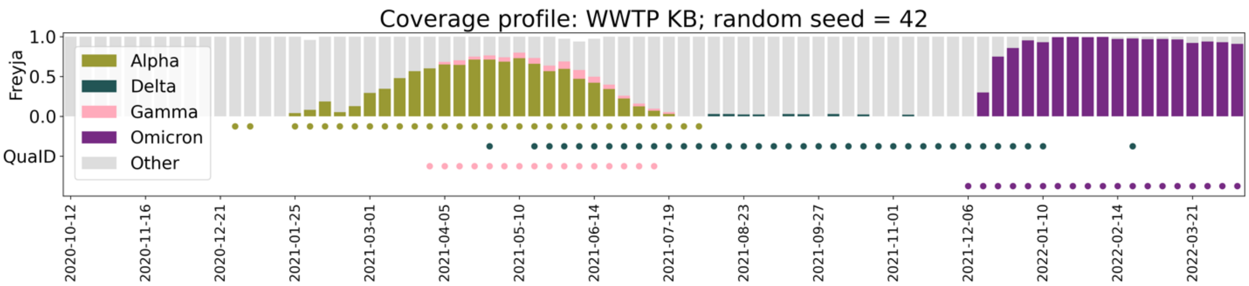

Supplementary Figure 20. (random seed 42) Freyja relative abundance estimates and QuaID detection signal on simulated data from GenBank (USA/TX) with the coverage profile based on the WWTP KB (mutations resampled as Bernoulli trials). Freyja is unable to detect the Delta VoC. QuaID robustly detects all VoCs with earlier detections of Alpha, Gamma, and Omicron variants.

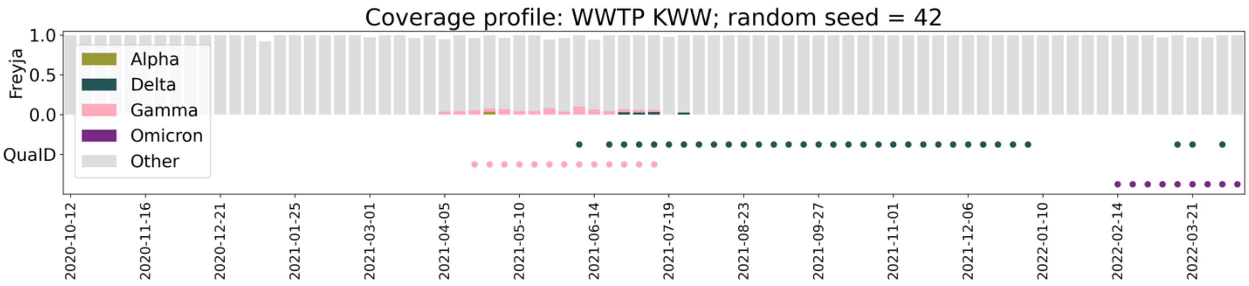

Supplementary Figure 21. (random seed 42) Freyja relative abundance estimates and QuaID detection signal on simulated data from GenBank (USA/TX) with the coverage profile based on the WWTP KWW (mutations resampled as Bernoulli trials). Freyja is any VoCs except the Gamma variant. QuaID detection suffers from drops in signal for Omicron VoC, and inability to detect Alpha variant.

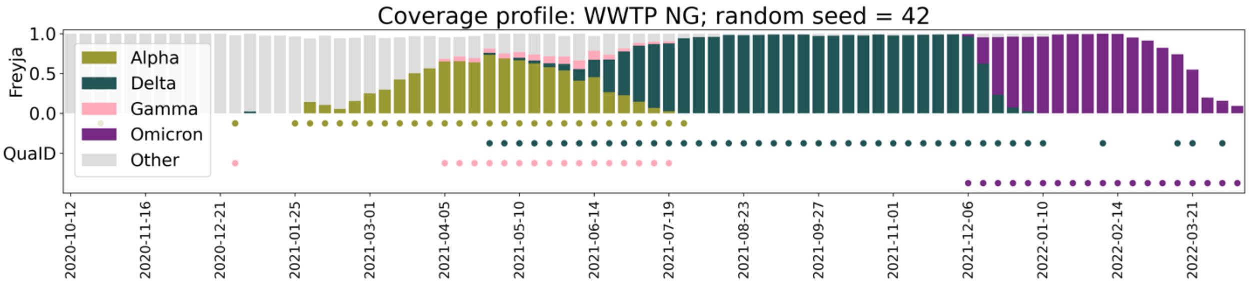

Supplementary Figure 22. (random seed 42) Freyja relative abundance estimates and QuaID detection signal on simulated data from GenBank (USA/TX) with the coverage profile based on the WWTP NG (mutations resampled as Bernoulli trials). Both Freyja and QuaID show robust detection patterns for all VoCs. QuaID detects presence of Alpha, Delta, Gamma, and Omicron VoCs at least one week earlier than Freyja.

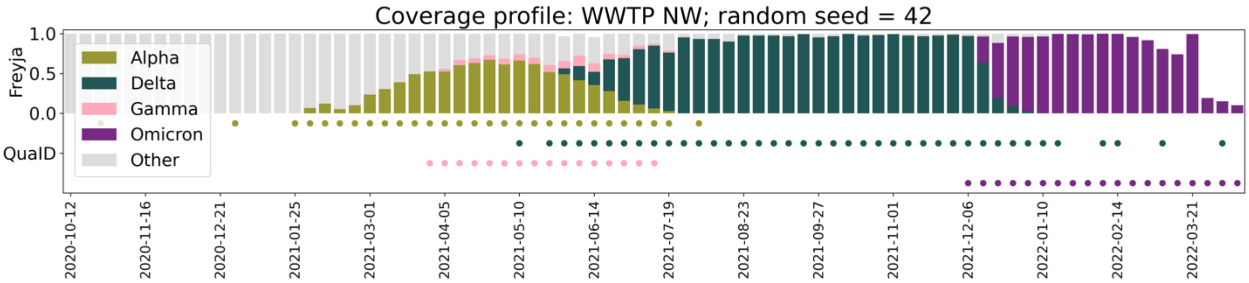

Supplementary Figure 23. (random seed 42) Freyja relative abundance estimates and QuaID detection signal on simulated data from GenBank (USA/TX) with the coverage profile based on the WWTP NW (mutations resampled as Bernoulli trials). Both Freyja and QuaID show robust detection patterns for all VoCs. QuaID detects presence of Alpha, Delta, Gamma, and Omicron VoCs at least one week earlier than Freyja.

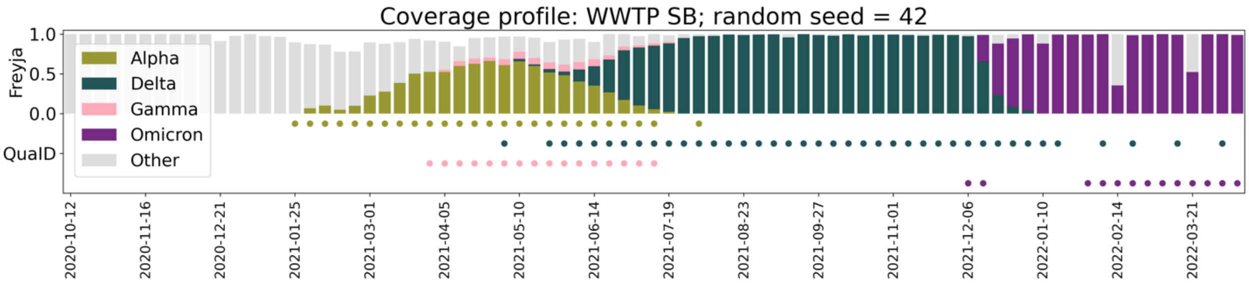

Supplementary Figure 24. (random seed 42) Freyja relative abundance estimates and QuaID detection signal on simulated data from GenBank (USA/TX) with the coverage profile based on the WWTP SB (mutations resampled as Bernoulli trials). Both Freyja and QuaID show robust detection patterns for all VoCs. QuaID detects presence of Alpha, Delta, Gamma, and Omicron VoCs at least one week earlier than Freyja, a minor drop in detection signal for Omicron occurs.

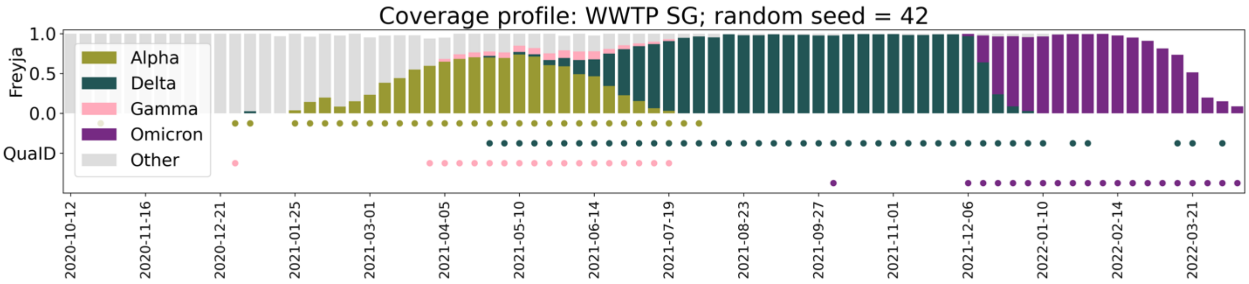

Supplementary Figure 25. (random seed 42) Freyja relative abundance estimates and QuaID detection signal on simulated data from GenBank (USA/TX) with the coverage profile based on the WWTP SG (mutations resampled as Bernoulli trials). Both Freyja and QuaID show robust detection patterns for all VoCs. QuaID detects presence of Alpha, Delta, Gamma, and Omicron VoCs at least one week earlier than Freyja.

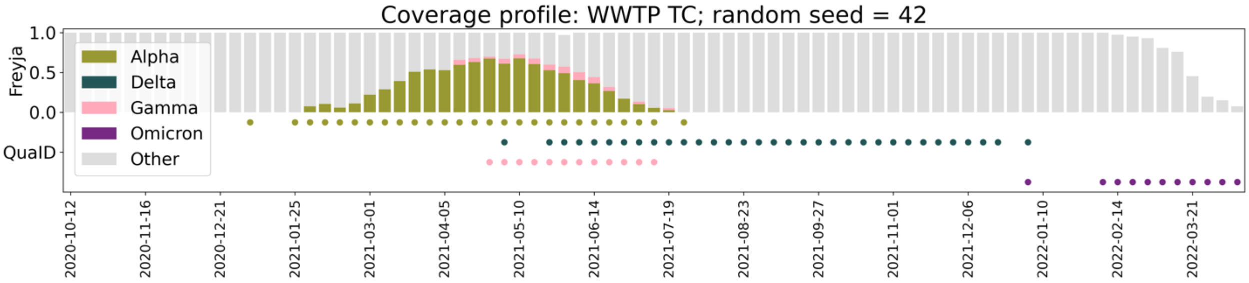

Supplementary Figure 26. (random seed 42) Freyja relative abundance estimates and QuaID detection signal on simulated data from GenBank (USA/TX) with the coverage profile based on the WWTP TC (mutations resampled as Bernoulli trials). Freyja is unable to detect Delta and Omicron VoCs. QuaID detects presence of Alpha, Delta, Gamma, and Omicron VoC, but has a drop in detection signal for Omicron.

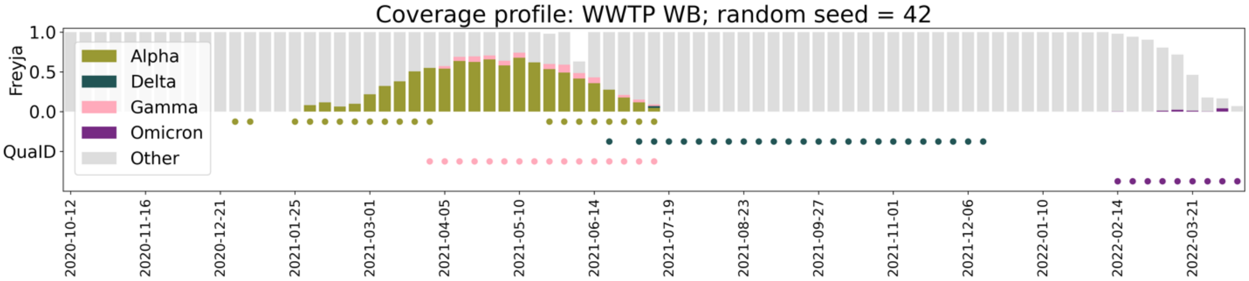

Supplementary Figure 27. (random seed 42) Freyja relative abundance estimates and QuaID detection signal on simulated data from GenBank (USA/TX) with the coverage profile based on the WWTP WB (mutations resampled as Bernoulli trials). Freyja is unable to detect Delta and Omicron VoCs. QuaID detects presence of Alpha, Delta, Gamma, and Omicron VoC, but has a drop in detection signal for Omicron.

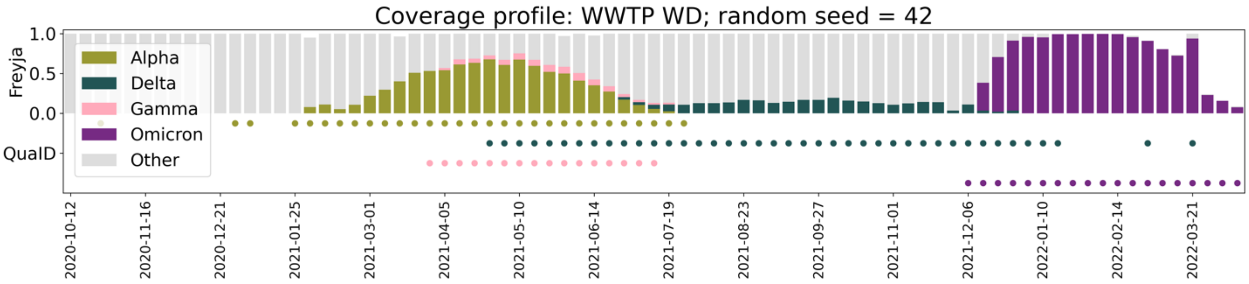

Supplementary Figure 28. (random seed 42) Freyja relative abundance estimates and QuaID detection signal on simulated data from GenBank (USA/TX) with the coverage profile based on the WWTP WD (mutations resampled as Bernoulli trials). Both Freyja and QuaID show robust detection patterns for all VoCs. QuaID detects presence of Alpha, Delta, Gamma, and Omicron VoCs at least one week earlier than Freyja.

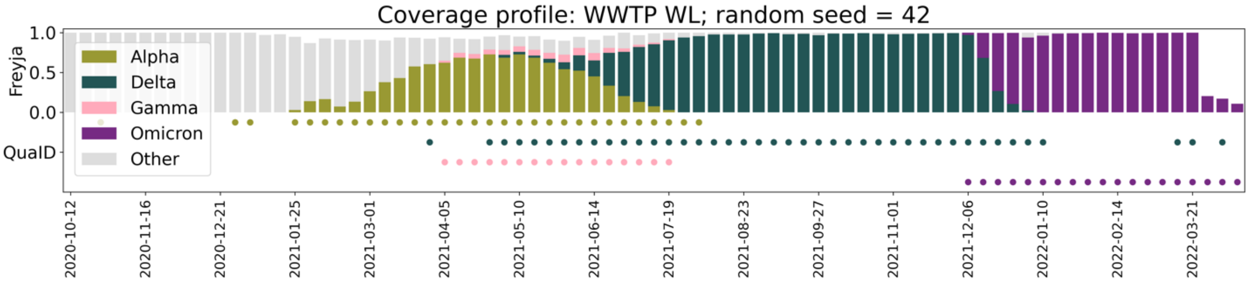

Supplementary Figure 29. (random seed 42) Freyja relative abundance estimates and QuaID detection signal on simulated data from GenBank (USA/TX) with the coverage profile based on the WWTP WL (mutations resampled as Bernoulli trials). Both Freyja and QuaID show robust detection patterns for all VoCs. QuaID detects presence of Alpha, Delta, Gamma, and Omicron VoCs at least one week earlier than Freyja.

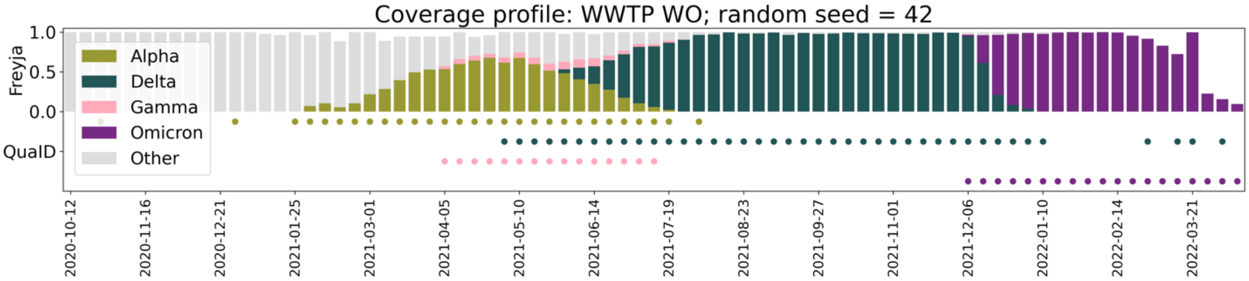

Supplementary Figure 30. (random seed 42) Freyja relative abundance estimates and QuaID detection signal on simulated data from GenBank (USA/TX) with the coverage profile based on the WWTP WO (mutations resampled as Bernoulli trials). Both Freyja and QuaID show robust detection patterns for all VoCs. QuaID detects presence of Alpha, Delta, Gamma, and Omicron VoCs at least one week earlier than Freyja.

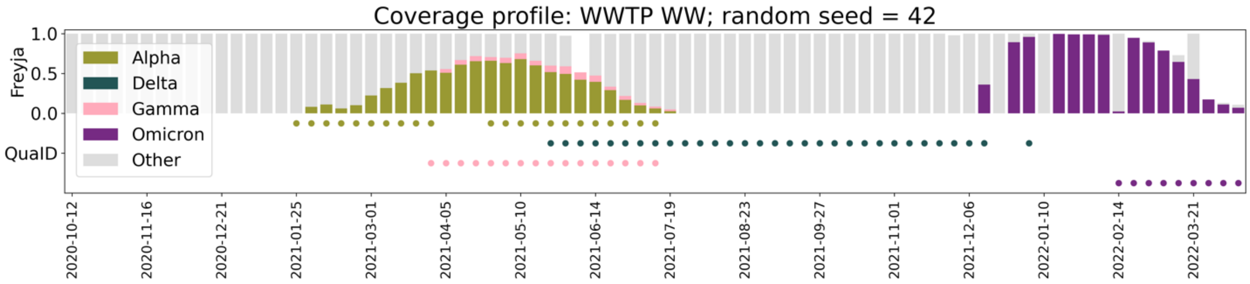

Supplementary Figure 31. (random seed 42) Freyja relative abundance estimates and QuaID detection signal on simulated data from GenBank (USA/TX) with the coverage profile based on the WWTP WB (mutations resampled as Bernoulli trials). Freyja is unable to detect Delta VoC. QuaID detects presence of Alpha, Delta, Gamma, and Omicron VoC, but has a drop in detection signal for Omicron.

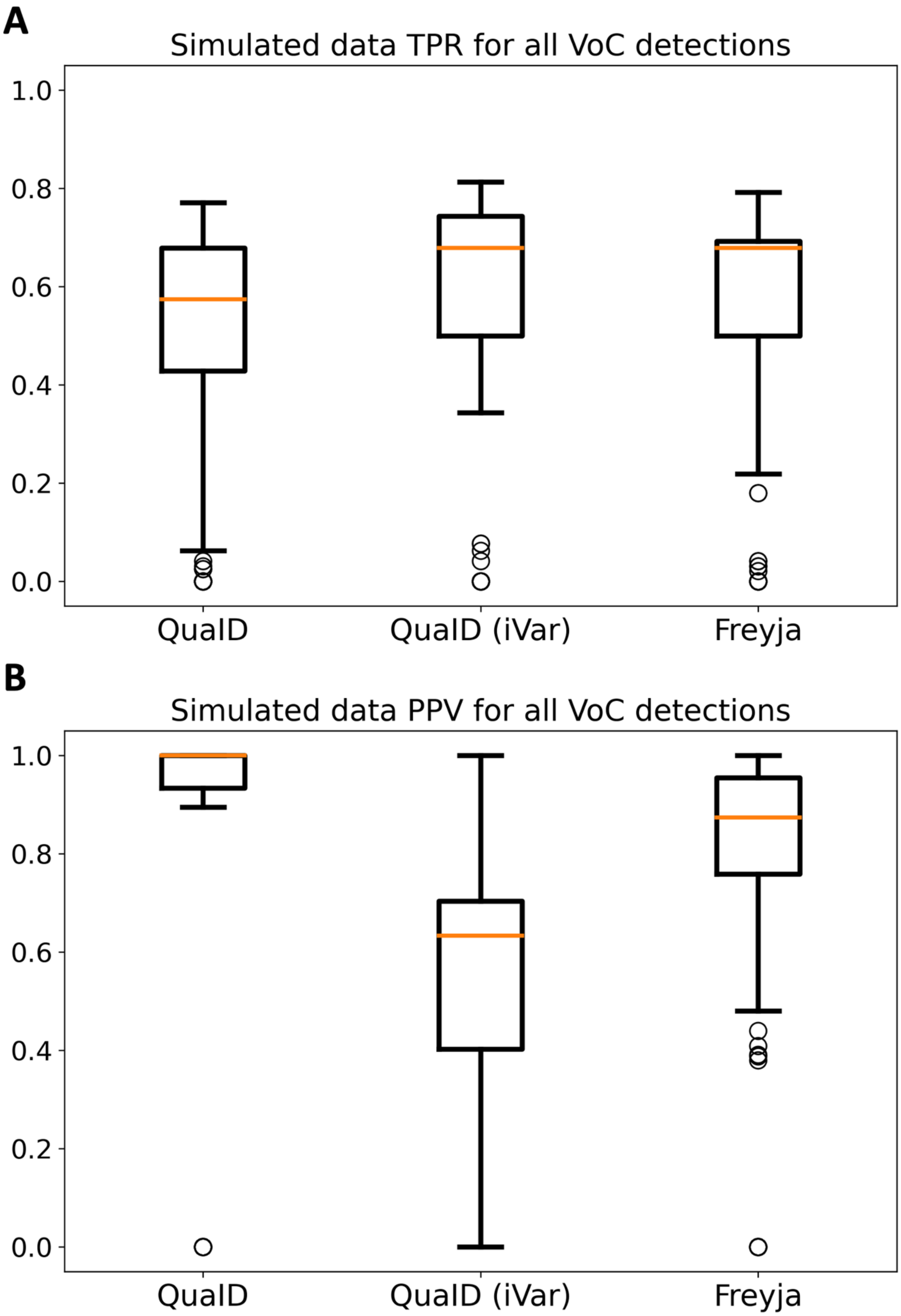
A
Simulated data TPR for all VoC detections
1.0
0.8
0.6
0.4
0.2
0.0
QualD
QualD (iVar)
Freyja
B
Simulated data PPV for all VoC detections
1.0
0.8
0.6
0.4
0.2
0.0
QualD
QualD (iVar)
Freyja

Supplementary Figure 32. (random seeds 17, 42) Recall (A) and precision (B) values obtained by QuaID, QuaID (iVar), and Freyja on simulated samples (n=8,112) with templates from the week of 12/16/2021 (see Methods protocol (c)). Orange line indicates median, bounds of the box are given by first and third quartiles, and the whiskers are extending the box by 1.5x inter-quartile range. QuaID (iVar) and Freyja achieve highest median recall (QuaID (iVar): 0.678, Freyja: 0.678), followed by QuaID with combined variant calls (QuaID: 0.574). QuaID obtains highest median precision (1.0), followed by Freyja (0.873), and QuaID (iVar) (0.633). In this figure QuaID (iVar) refers to using QuaID with variant calls obtained by running iVar, i.e. the variant calls are not a consensus between iVar and LoFreq.
