# Supplementary material for: Enabling accurate and early detection of recently emerged SARS-CoV-2 variants of concern in wastewater
Source: Nat Commun. 2023 May 17;14:2834. doi: 10.1038/s41467-023-38184-3 (PMC10191095; doi:10.1038/s41467-023-38184-3)
Supplement: Supplementary file 3 — Description of Additional Supplementary Files [file 41467_2023_38184_MOESM3_ESM.pdf]

Title: Supplementary Data 1

Description: QuaID output reporting date, wastewater treatment plant, observed allele frequency of quasi-unique mutations, and the corresponding variant of concern. This file includes reporting for Alpha, Delta, Gamma, Omicron, and BA.1-5 variants.
