# Supplementary material for: Enabling accurate and early detection of recently emerged SARS-CoV-2 variants of concern in wastewater
Source: Nat Commun. 2023 May 17;14:2834. doi: 10.1038/s41467-023-38184-3 (PMC10191095; doi:10.1038/s41467-023-38184-3)
Supplement: Supplementary file 5 — Reporting Summary [file 41467_2023_38184_MOESM5_ESM.pdf]

nature portfolio

Corresponding author(s): Todd J. Treangen
Last updated by author(s): May 8, 2023

Reporting Summary

Nature Portfolio wishes to improve the reproducibility of the work that we publish. This form provides structure for consistency and transparency in reporting. For further information on Nature Portfolio policies, see our Editorial Policies and the Editorial Policy Checklist.

Statistics

For all statistical analyses, confirm that the following items are present in the figure legend, table legend, main text, or Methods section.

n/a	Confirmed	
☐	☒	The exact sample size (n) for each experimental group/condition, given as a discrete number and unit of measurement
☒	☐	A statement on whether measurements were taken from distinct samples or whether the same sample was measured repeatedly
☒	☐	The statistical test(s) used AND whether they are one- or two-sided<br>*Only common tests should be described solely by name; describe more complex techniques in the Methods section.*
☒	☐	A description of all covariates tested
☒	☐	A description of any assumptions or corrections, such as tests of normality and adjustment for multiple comparisons
☐	☒	A full description of the statistical parameters including central tendency (e.g. means) or other basic estimates (e.g. regression coefficient) AND variation (e.g. standard deviation) or associated estimates of uncertainty (e.g. confidence intervals)
☒	☐	For null hypothesis testing, the test statistic (e.g. F, t, r) with confidence intervals, effect sizes, degrees of freedom and P value noted<br>*Give P values as exact values whenever suitable.*
☒	☐	For Bayesian analysis, information on the choice of priors and Markov chain Monte Carlo settings
☒	☐	For hierarchical and complex designs, identification of the appropriate level for tests and full reporting of outcomes
☒	☐	Estimates of effect sizes (e.g. Cohen's d, Pearson's r), indicating how they were calculated

Our web collection on statistics for biologists contains articles on many of the points above.

Software and code

Policy information about availability of computer code

Data collection	No software was used in the data collection process.
Data analysis	FastQC (v0.11.9), BBDuk (38.90), BWA MEM (0.7.17), iVar (1.3.1), LoFreq (2.1.5), vdb (2.7), Freyja (1.3.4), ART (MountRainier, 2016-06-05); custom Python code developed (QuaID) is publicly available on GitLab: https://gitlab.com/treangenlab/quaid

For manuscripts utilizing custom algorithms or software that are central to the research but not yet described in published literature, software must be made available to editors and reviewers. We strongly encourage code deposition in a community repository (e.g. GitHub). See the Nature Portfolio guidelines for submitting code & software for further information.

Data

Policy information about availability of data

All manuscripts must include a data availability statement. This statement should provide the following information, where applicable:

- Accession codes, unique identifiers, or web links for publicly available datasets
- A description of any restrictions on data availability
- For clinical datasets or third party data, please ensure that the statement adheres to our policy

Sequencing data used is available at SRA via BioProject accession PRJNA796340, SARS-CoV-2 genomes used in the construction of the simulated datasets are available on GenBank (https://www.ncbi.nlm.nih.gov/labs/virus/vssi/#/virus?SeqType_s=Nucleotide&VirusLineage_ss=taxid:2697049), SARS-CoV-2 multiple sequence alignments used for database construction, and their associated metadata are available from GISAID (https://gisaid.org).

Human research participants

Policy information about studies involving human research participants and Sex and Gender in Research.

Reporting on sex and gender	N/A
Population characteristics	N/A
Recruitment	N/A
Ethics oversight	N/A

Note that full information on the approval of the study protocol must also be provided in the manuscript.

Field-specific reporting

Please select the one below that is the best fit for your research. If you are not sure, read the appropriate sections before making your selection.

☐ Life sciences ☐ Behavioural & social sciences ☒ Ecological, evolutionary & environmental sciences

For a reference copy of the document with all sections, see nature.com/documents/nr-reporting-summary-flat.pdf

Ecological, evolutionary & environmental sciences study design

All studies must disclose on these points even when the disclosure is negative.

Study description	The study collected wastewater samples from 39 wastewater treatment plants around Houston metropolitan area. Samples were used to extract viral RNA and amplicon sequencing of SARS-CoV-2 was performed. Resulting amplicons were then then sequenced and the paired-end Illumina sequencing data were used for the downstream computational analyses. Downstream analyses included read mapping, variant calling and subsequent variant of concern detection which was performed using the code developed as the part of the study. Total number of samples analyzed is 2,637. Additionally simulated samples were generated (n=32,448). For empirical samples no replicates were created, but a negative and positive controls for SARS-CoV-2 were sequenced in parallel to detect any possible sample cross-contamination. The simulated data has been generated with two separate random seeds (17, 42).
Research sample	Time-weighted composite samples of raw wastewater were collected every 1 h for 24 h from the influent of the wastewater treatment plants. SARS-CoV-2 was concentrated in wastewater samples using electronegative filtration. RNA extraction was performed using a Chemagic™ Prime Viral DNA/RNA 300 Kit H96 (Chemagic, CMG-1433, PerkinElmer) with the PerkinElmer viral RNA/DNA purification protocol and reagents. Sequencing was performed by the Houston Health Department laboratory and several different sequencing kits, library preparation kits, and primer panels were used to amplify SARS-CoV-2 genomes in wastewater samples (Supplementary Table 2). cDNA was generated using 8 - 11 µL RNA extract via reverse transcription using the Superscript IV first-strand synthesis system (ThermoFisher Scientific, 18091050) following the manufacturer's protocol. SARS-CoV-2 genome enrichment via multiplexing PCR was conducted.<br><br>Each sample represents collection of viral RNA fragments found in the wastewater in the geographic region corresponding to the wastewater treatment plant. Due SARS-CoV-2 shedding into infected individuals waste products, samples are meant to represent a population snapshot of currently circulating SARS-CoV-2 virus. Since we have performed amplicon based sequencing each resulting sample should only contain reads from the viral RNA fragments of SARS-CoV-2. Thus, the term sample in our study refers to a set of sequencing reads obtained from SARS-CoV-2 genomic fragments found in the municipal wastewater.
Sampling strategy	Untreated wastewater samples were collected from the influent channel using refrigerated 24-hour composite samplers at each wastewater treatment plant. The autosamplers collected an aliquot of wastewater (200 mL) every hour over 24 hours.
Data collection	Untreated wastewater samples were collected from the influent channel using refrigerated 24-hour composite samplers at each wastewater treatment plant. The autosamplers collected an aliquot of wastewater (200 mL) every hour over 24 hours. After sample collection, samples were placed on ice and transported to Houston Water's laboratory, aliquoted into 250 mL and 500 mL bottles, and transported on ice to Rice University (Rice) for processing. Houston Water provided the influent flowrates for each WWTP corresponding to the 24-hour sampling period.
Timing and spatial scale	Samples were collected weekly between February 23rd, 2021 and May 5th, 2022 from 39 wastewater treatment plants in Houston metropolitan area covering 580 miles2 and servicing approximately 2.3 million people. We included all wastewater treatment plants in the city of Houston in the study in order to investigate the emergence of variants of concern within a large metropolitan area. The sampling schedule was determined based on the available resources and the goal of providing dense longitudinal sampling.
Data exclusions	No data was excluded from analysis.
Reproducibility	All computational analyses were performed using fixed versions of software, and reproducibility is ensured by providing the details on versions of the third party software and sharing the open source for the software developed in the study. Replication of the QuaID analysis of the empirical data was performed weekly for the duration of the study.

Randomization	Samples were not randomized, since the study did not investigate any correlations based on any grouping of the samples.
Blinding	There was no blinding for the empirical samples, since the a priori viral strain compositions of the empirical samples are unknown. For the simulated data, generated simulated reads contained no provenance information for the corresponding origin genomes. Thus, no metadata leakage in the simulation was ensured.

Did the study involve field work? ☐ Yes ☒ No

Reporting for specific materials, systems and methods

We require information from authors about some types of materials, experimental systems and methods used in many studies. Here, indicate whether each material, system or method listed is relevant to your study. If you are not sure if a list item applies to your research, read the appropriate section before selecting a response.

Materials & experimental systems

n/a	Involved in the study	
☒	☐	Antibodies
☒	☐	Eukaryotic cell lines
☒	☐	Palaeontology and archaeology
☒	☐	Animals and other organisms
☒	☐	Clinical data
☒	☐	Dual use research of concern

Methods

n/a	Involved in the study	
☒	☐	ChIP-seq
☒	☐	Flow cytometry
☒	☐	MRI-based neuroimaging
